# Silent Sigmoid Colon Diverticular Perforation: A Case Report

**DOI:** 10.7759/cureus.14900

**Published:** 2021-05-08

**Authors:** Sifullah Bashar, Ali F Al Sbihi, Nouraldeen Manasrah, Ahmad J Chaudhary, Sana Iqbal

**Affiliations:** 1 Internal Medicine, Detroit Medical Center/Wayne State University Sinai-Grace Hospital, Detroit, USA; 2 Internal Medicine, Detroit Medical Center Sinai-Grace Hospital, Detroit, USA; 3 Internal Medicine, Detroit Medical Center, Detroit, USA

**Keywords:** sigmoid diverticulitis, sigmoid diverticulosis, silent pneumoperitoneum, free air under the diaphragm, acute serositis

## Abstract

Colonic perforation is associated with high mortality rates, and it requires prompt diagnosis and intervention to ensure favorable patient outcomes. The condition usually presents with typical peritoneal signs and symptoms, but atypical presentations can be a diagnostic challenge. In this report, we present a case of sigmoid diverticulosis perforation in an elderly patient who had no symptoms after the perforation developed. This case highlights the importance of detailed history, physical examination, and a low threshold of suspicion in patients with risk factors for atypical presentations.

## Introduction

Diverticular perforation can be asymptomatic in elderly patients. Clinicians should maintain a high index of suspicion for bowel perforation in the elderly, especially those who have a history of diverticulosis. Detailed history including any history of immunosuppressive or anti-inflammatory medications, physical examination, laboratory tests, and imaging studies must be appropriately pursued to avoid serious complications including death. In this report, we discuss a case of an 88-year-old woman who showed no symptoms even after the perforation developed. Her case was promptly and successfully managed.

## Case presentation

An 88-year-old African American female with a past medical history of hypertension, primary hypothyroidism that was controlled on medications, and diverticulosis was admitted to the hospital with hematochezia and perianal pain. Her mentation was within normal limits and she denied any symptoms of fever, nausea, vomiting, abdominal pain, or change in bowel habits. Drug history included furosemide, indapamide, and levothyroxine. On presentation, she had a blood pressure of 153/86 mmHg, heart rate (HR) of 99 beats per minute (BPM), respiratory rate (RR) of 17 breaths per minute, a temperature of 37.3 °C, and oxygen saturation of 99% on room air. Her abdomen was non-distended, soft, non-tender, and had normal bowel sounds on physical examination.

Laboratory findings showed a white blood cell (WBC) count of 8,300 cells/ul (normal range: 4,500-11,000 cells/ul), lactic acid of 1.4 mMol/L (normal range: 0.4-2.0 mMol/L), hemoglobin of 9.6 g/dl (baseline: 9-10 g/dl) (normal range: 14-17 g/dl). Platelets were 405,000/ul (normal range: 150,000-400,000/ul). Mean corpuscular volume (MCV) was 93.8 fL (normal range: 80-100 fL), mean corpuscular hemoglobin (MCH) was 28.5 pg (27.5 and 33.2 pg), mean corpuscular hemoglobin concentration (MCHC) was 30.4 g/dL (normal range: 31-35 g/dL), and red cell distribution width (RDW) was 16.3% (normal range: 11.8-14.5%). The absolute neutrophil count was 6,600 cells/ul (normal range: 1,600-7,000 cells/ul). Bands were 2,500 cells/ul (normal range: 0-500 cells/ul). Thyroid-stimulating hormone (TSH) was 2.85 milliunits/L (normal range: 0.5-5 milliunits/L). Urinalysis showed cloudy urine with 3+ leukocyte esterase, positive nitrites, and 3+ bacteria; hence, the patient was treated conservatively with 1 gm ceftriaxone for urinary tract infection (UTI). An electrocardiogram (EKG) and a chest X-ray (CXR) did not show any abnormalities. Angiography was performed by interventional radiology and it was negative for active bleeding. Colonoscopy was performed and revealed severe diverticulosis, which was similar to the last colonoscopy performed two months ago during a previous admission. The gastrointestinal surgery team recommended a total colectomy but the patient refused it. The patient did not have any further bleeding episodes during the hospital stay. Hemodynamic parameters remained stable, and her hemoglobin remained stable at its baseline.

**Figure 1:**
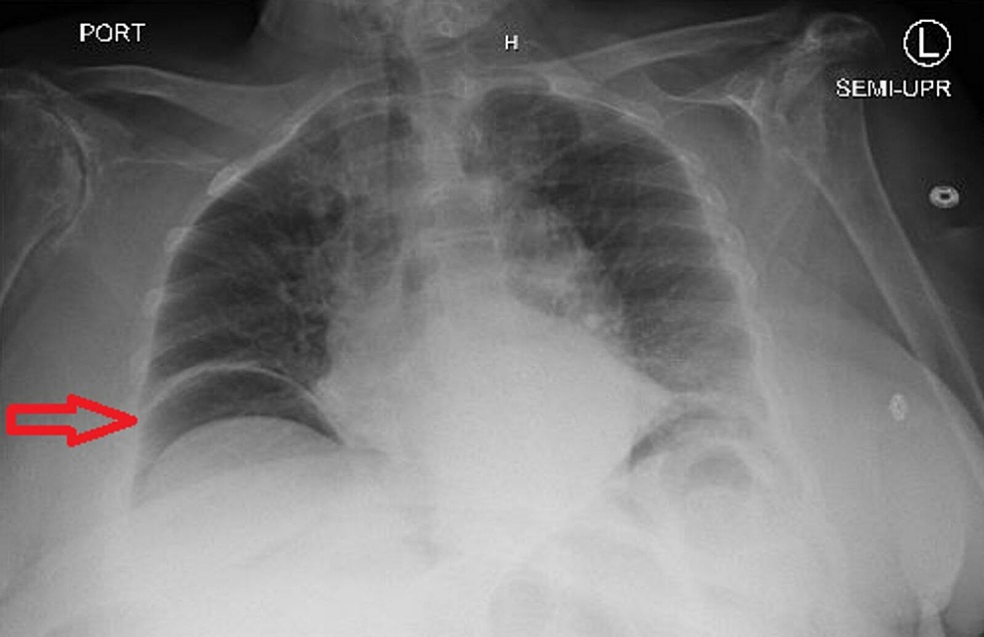
CXR showing air under diaphragm’s right dome (arrow) CXR: chest X-ray

**Figure 2:**
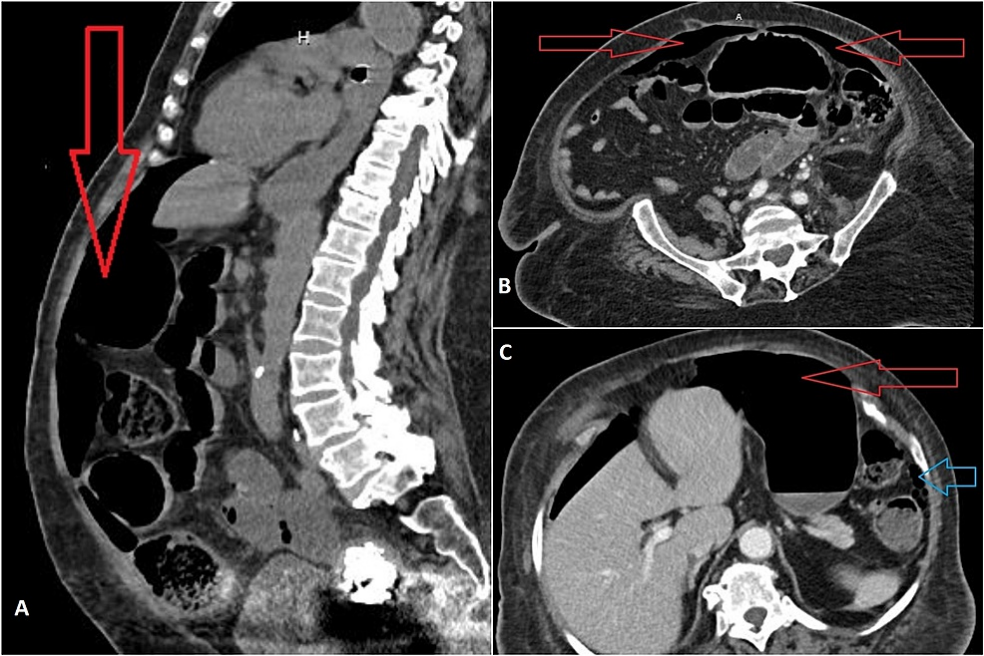
Abdominopelvic CT scan A: sagittal view. B and C: axial views. Red arrows indicate pneumoperitoneum while the blue arrow indicates gas in the pericolic fat suggestive of colonic perforation CT: computed tomography

Subacute rehabilitation discharge was planned; however, on the fifth day of admission, the patient developed a fever of 39.3 °C; her HR was found to be 125 BPM, and the BP remained stable at 132/82 mmHg. The patient continued to remain alert and oriented, and she did not have any abdominal pain, hematochezia, hematemesis, nausea, vomiting, or change in bowel habits. The abdomen was still non-distended, soft, and non-tender, but there were no bowel sounds. Breathing sounds were diminished on the right lower chest, and hence a CXR was obtained, which revealed free gas under the diaphragm’s right dome suggesting a perforated viscus (Figure 1). An abdominopelvic contrast-enhanced CT scan was performed and revealed evidence of large pneumoperitoneum and inflammatory stranding involving the distal descending and sigmoid colon suggestive of acute diverticulitis (Figure 2). The WBC count continued to be within normal limits (10,300 cells/ul) and hemoglobin was found to be 8.9 g/dl (no significant drop).

**Figure 3:**
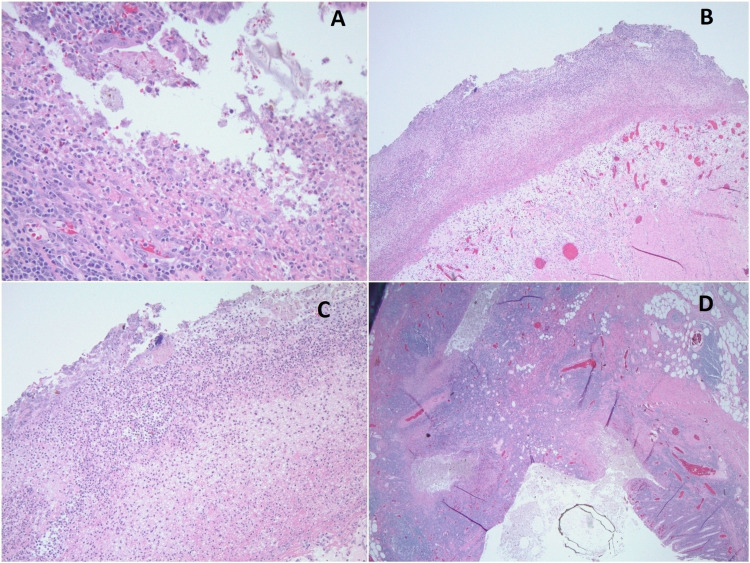
Pathology images using hematoxylin and eosin (H&E) staining A: acute diverticulitis B: low power field microscopy showing acute serositis (total magnification is 100x using 10x lens) C: high power field showing acute serositis with serosa’s fibrinopurulent exudates (total magnification is 400x using 40x lens) D: acute diverticulitis with marked transmural inflammation and surface mucosal ulceration

Exploratory laparotomy, sigmoid colectomy, and end-colostomy were emergently performed by gastrointestinal surgery, and tissue specimen was sent for pathological examination. The operative and pathological findings were consistent with a Hinchey stage IV perforation [1], associated with fecal peritonitis (Figure 3). The patient was treated with ceftriaxone and metronidazole. In the postoperative period, the patient remained tachycardic with HR up to 130 BPM, and tachypneic with RR up to 30 breaths per minute; however, other vital signs were normal. The abdomen was soft and non-tender, and the wound was dry and clean with no complications. Clinically, the patient's condition continued to improve, and the vital signs and the WBC count gradually returned to normal limits. When the patient was completely asymptomatic, she was discharged to a sub-acute rehabilitation, and by that time, she had already finished a two-week antibiotic course. Her blood cultures had remained negative during the entire hospital stay.

## Discussion

Colonic diverticulosis, which is defined as the presence of multiple uninflamed colonic diverticula, is a gastrointestinal disease that is associated with a huge financial burden and expense among the United States population as per a recent study [2]. It is usually asymptomatic, but symptoms can involve mild abdominal pain, typically in the left lower abdominal quadrant, and severe peritonitis [3]. Diverticular perforation leading to peritonitis is the most dangerous and severe complication of the disease [3].

Classically, pneumoperitoneum secondary to bowel perforation presents with typical symptoms including fever, nausea, and severe abdominal pain, in addition to clinical signs of peritonitis on examination. In rare cases, pneumoperitoneum can present silently without clinical signs of illness; and such instances have been described in case reports [4-7]. Perforation of the colon is associated with high morbidity and mortality, and hence it requires early diagnosis. However, the diagnosis of perforation arising from atypical causes can be challenging.

Corticosteroid use has been described as a cause of spontaneous diverticular perforation [8-10]. The immunosuppressive effect of corticosteroids results in the impaired ability to contain the perforation during the early stages [11]. Also, the use of non-steroidal anti-inflammatory drugs (NSAIDs) has been associated with an increased risk of perforation through many reported factors like epithelial cells’ damage, reduced mucin secretion [11], and reduced synthesis of prostacyclin [12]. Studies have described an increased likelihood of silent bowel perforation during corticosteroid treatment, possibly due to anti-inflammatory effects that disrupt the physiological healing process, thereby masking the symptoms [4].

Additionally, advanced age, opioid use, abdominal infection, and radiation therapy contributing to tissue damage are among the most important factors that increase the risk of developing silent pneumoperitoneum. Pathologically, rapid closure of the leaking diverticulum and the transmural passage of air through a thin-walled diverticulum are among the factors that are reported to contribute to spontaneous and asymptomatic pneumoperitoneum [5].

A recent hypothesis regarding the association between disease-modifying antirheumatic drugs (DMARDs) and spontaneous diverticular perforation has been proposed in the literature, but a definitive association has not been established yet. A given example of this association is a case of a rheumatoid arthritis patient on methotrexate who had a spontaneous diverticular perforation [6].

An example of the association between silent pneumoperitoneum and corticosteroids/DMARDs was seen in the case of a 57-year-old male who had pemphigus vulgaris treated with immunosuppressive therapy of steroids and azathioprine; he had recurrent sigmoid colonic perforation but did not present with an acute abdomen. The authors concluded that both steroids and azathioprine's immunosuppressive effects may have completely obscured the peritonitis inflammatory signs, resulting in the absence of typical presenting symptoms [7].

## Conclusions

Pneumoperitoneum due to bowel perforation is a life-threatening emergency that requires urgent surgical evaluation and intervention. Our patient developed asymptomatic diverticular perforation on her fifth day of hospital admission; we concluded that her only risk factor was her advanced age, and we ruled out other risk factors for such a presentation. We would like to emphasize the importance of recognizing atypical presentations of diverticular perforation. Although it was not the case in our patient, it is crucial to know that in rare cases, immunosuppressive or anti-inflammatory agents may impair inflammatory response, and patients may consequently have minimal or complete lack of symptoms and signs. In the setting of bowel disorders with risk factors for bowel perforation, a high index of suspicion is required even in cases with a benign physical examination.
